# Calibrating Wrist-Worn Accelerometers for Physical Activity Assessment in Preschoolers: Machine Learning Approaches

**DOI:** 10.2196/16727

**Published:** 2020-08-31

**Authors:** Shiyu Li, Jeffrey T Howard, Erica T Sosa, Alberto Cordova, Deborah Parra-Medina, Zenong Yin

**Affiliations:** 1 The University of Texas Health Science Center at San Antonio San Antonio, TX United States; 2 Department of Public Health The University of Texas at San Antonio San Antonio, TX United States; 3 Department of Kinesiology The University of Texas at San Antonio San Antonio, TX United States; 4 Department of Mexican American and Latina/o Studies The University of Texas at Austin Austin, TX United States

**Keywords:** preschoolers, accelerometer, physical activity, obesity, machine learning

## Abstract

**Background:**

Physical activity (PA) level is associated with multiple health benefits during early childhood. However, inconsistency in the methods for quantification of PA levels among preschoolers remains a problem.

**Objective:**

This study aimed to develop PA intensity cut points for wrist-worn accelerometers by using machine learning (ML) approaches to assess PA in preschoolers.

**Methods:**

Wrist- and hip-derived acceleration data were collected simultaneously from 34 preschoolers on 3 consecutive preschool days. Two supervised ML models, receiver operating characteristic curve (ROC) and ordinal logistic regression (OLR), and one unsupervised ML model, k-means cluster analysis, were applied to establish wrist-worn accelerometer vector magnitude (VM) cut points to classify accelerometer counts into sedentary behavior, light PA (LPA), moderate PA (MPA), and vigorous PA (VPA). Physical activity intensity levels identified by hip-worn accelerometer VM cut points were used as reference to train the supervised ML models. Vector magnitude counts were classified by intensity based on three newly established wrist methods and the hip reference to examine classification accuracy. Daily estimates of PA were compared to the hip-reference criterion.

**Results:**

In total, 3600 epochs with matched hip- and wrist-worn accelerometer VM counts were analyzed. All ML approaches performed differently on developing PA intensity cut points for wrist-worn accelerometers. Among the three ML models, k-means cluster analysis derived the following cut points: ≤2556 counts per minute (cpm) for sedentary behavior, 2557-7064 cpm for LPA, 7065-14532 cpm for MPA, and ≥14533 cpm for VPA; in addition, k-means cluster analysis had the highest classification accuracy, with more than 70% of the total epochs being classified into the correct PA categories, as examined by the hip reference. Additionally, k-means cut points exhibited the most accurate estimates on sedentary behavior, LPA, and VPA as the hip reference. None of the three wrist methods were able to accurately assess MPA.

**Conclusions:**

This study demonstrates the potential of ML approaches in establishing cut points for wrist-worn accelerometers to assess PA in preschoolers. However, the findings from this study warrant additional validation studies.

## Introduction

Accelerometry has been widely accepted as the gold standard to measure physical activity (PA) in free-living settings [1,2] including preschools [3]. Triaxial accelerometers can record the magnitude of accelerations from three movement axes and convert accelerations to vector magnitude counts over a given user-specified cycling period (epoch) [4]. Counts are translated into biologically meaningful PA volume and intensity levels using pre-established cut points for sedentary behavior, light physical activity (LPA), moderate physical activity (MPA), and vigorous physical activity (VPA) [3]. Although; traditionally, gold standard cut points are established using data derived from accelerometers placed on the right hip of the body [1,2], recent revolutions have focused on acceleration data from wrist-worn accelerometers for increased protocol compliance of study participants, better sensitivity to detect certain types of movements, and sleep measurement [5-9]. Fairclough et al [10] found that wrist-worn accelerometers had at least 10% higher compliance rate than hip-worn ones, regardless of the data processing criteria in school-age children. They also reported that wrist-worn accelerometers had a much lower study drop-off rate compared to hip-worn ones, regardless of the number of monitoring days. Thus, the wrist, instead of the hip, might be an ideal accelerometer placement site for preschoolers.

However, cut points from wrist-derived data are sparse for preschool-age children [11,12]. Johansson and colleagues [13,14] conducted the only studies that established wrist-referenced cut points for sedentary behavior and moderate and vigorous physical activity (MVPA) in preschoolers. Using direct observation of structured and free-play activities as the ground truth activities, hip- and wrist-derived cut points yielded comparable accuracy and validity of the observed activities. Nevertheless, Johansson [15] cut points did not differentiate between LPA, MPA, and VPA, and have not been replicated by others.

Machine learning (ML) algorithms are increasingly being used to translate accelerometer outputs to meaningful PA metrics [16]. Recent studies have applied part of the accelerometry data to ML algorithms as the training set to build statistical models that can predict PA intensities from a new set of accelerometry data without explicit instructions [17]. Research has demonstrated the promising performance of ML techniques in combination with the use of wrist-derived acceleration data in predicting the type and intensity of activities, as well as activity energy expenditure with comparable overall predictive accuracies in adult populations [16,18,19].

Combining ML techniques and wrist-worn accelerometers may help address the low compliance caused by the challenges in wearing hip-worn accelerometers [20,21] and the difficulty in measuring the various levels of activity intensity given the unique nature of the sporadic and short-burst activity patterns in preschool-age children [22,23]. Therefore, the purposes of this study were (1) to develop the cut point values for sedentary behavior, LPA, MPA, and VPA based on wrist-derived acceleration data using multiple ML algorithms, and (2) to examine classification accuracy of PA intensity in comparison to hip-reference cut points [24] and previously established wrist-referenced cut points [25] in preschool-age children.

## Methods

### Recruitment

A total of 61 healthy children, aged 3-5 years, who were enrolled in a Head Start program (HS), were recruited to participate in the study in Fall 2018; in San Antonio, Texas. The HS is a federally funded program that serves children from low-income families through academic, health, nutrition, and family service programs [26]. Their parents/guardians signed an informed consent form before participation. Each participant received up to US $30 gift card for participating in the study. Children who were 3 years old at recruitment, were enrolled in the full-day HS program, and had no physical disabilities were eligible for the study.

This study was reviewed and approved by the Institutional Review Board of the University of Texas at San Antonio.

### Accelerometer Data Collection

**Figure 1:**
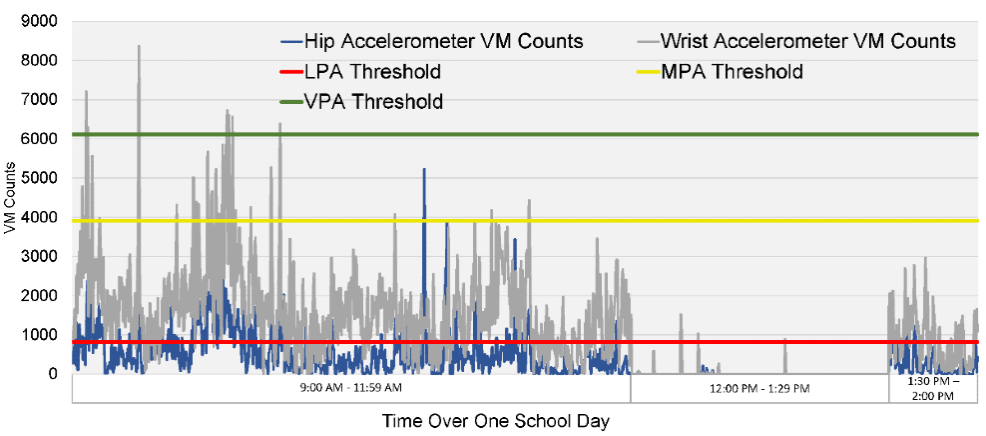
Visual presentation of the changes of wrist- and hip-worn accelerometer vector magnitude counts for one participant throughout a school day, with previously established hip-based physical activity level thresholds as defined by Butte et al. [23].

For 3 consecutive days, the children wore two triaxial accelerometers (ActiGraph model WGT3X-BT, ActiGraph, LLC) that collected raw accelerometry data at a sampling frequency of 30Hz (30 observations per second for each axis) from 9 AM to 2 PM, following a previously published protocol [27]. On the day of data collection, a trained research assistant placed one accelerometer on the nondominant wrist and the other one on the right hip of each child. For this study, raw accelerations were converted into 15-second epoch and thereafter collapsed to 60-second epochs. For this study, data were outputted as the vector magnitude (VM) counts, which is the square root of the sum of squares of each axis of acceleration data. Nonwear time was detected using the Choi wear time validation algorithm [28]. Participants with missing hip- or wrist-worn accelerometer epoch for more than 3 consecutive 5-hour days were excluded from the analysis. Accelerometer data processing was performed using ActiLife software (Version 6.13.3). Visual presentation of the accelerometer counts for one participant is shown in Figure 1.

### Hip- and Wrist-Reference Cut Points for Comparisons

The hip-reference cut points for assessing PA intensity were adopted from Butte et al [24] based on predicted energy expenditure from room calorimetry and doubly labeled water. The cut points were no more than 820 counts per minute (cpm) for sedentary behavior, 821-3908 cpm for LPA, 3909-6112 cpm for MPA, and greater than 6113 cpm for VPA for vector magnitude counts collected from ActiGraph hip-worn accelerometers during free-living activities in preschool-age children. The cut points reported by Butte and colleagues [13] are widely used and will be used as the gold-standard reference in this study. The cut points for sedentary behavior and MVPA from wrist-derived data captured during structured and free-play activities by Johansson et al [14] are the only available references for preschool-age children.

### Applications of Machine Learning Techniques

**Figure 2:**
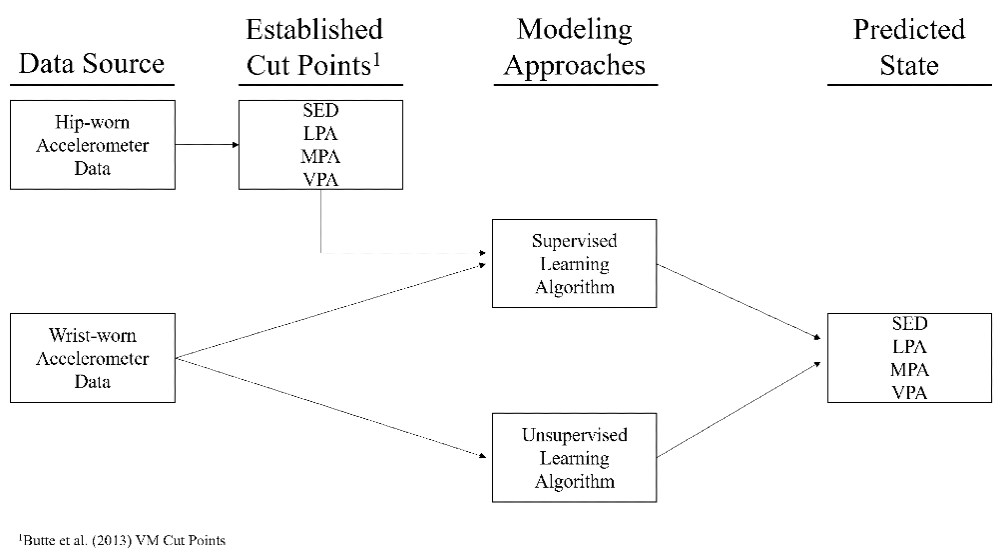
Modeling process diagram.

Three ML models, two supervised and one unsupervised, were used to establish three different sets of wrist-worn accelerometer VM cut points as the new wrist methods to assess PA in preschoolers (Figure 2). Supervised ML models learn from hip-identified PA of each epoch and produce an inferred function that maps the wrist accelerometer count to a PA category; unsupervised models read the underlying structure of the wrist-worn accelerometer counts and detect the PA level of each count value [29]. PA intensity levels identified using hip-worn accelerometer cut points by Butte et al [24] were used as the hip reference to train the supervised ML models. The two supervised ML techniques were receiver operating characteristic (ROC) analysis and the ordinal logistic regression (OLR) model. Since ROC analysis was designed to predict binary outcomes, it was run three times to establish the upper threshold for sedentary behavior and the lower thresholds for LPA and VPA [30]. After ROC analysis calculated and compared sensitivity values for all possible threshold values, we selected thresholds based on the minimum difference between sensitivity and specificity [31]. For the OLR method, after being trained by the PA intensity levels as predicted by the hip reference, the newly constructed model calculated and compared the probability of each VM count value being classified into different PA intensity levels and assigned each count to the PA level with the highest predicted probability. K-means cluster analysis was the unsupervised learning approach used to separate each 15-second epoch for each participant into four distinct clusters, based solely on the input VM count data [32-34]. The number of clusters (k=4) was determined a priori because the four activity states of sedentary behavior, LPA, MPA, and VPA were known. Sedentary behavior cut points for OLR and k-means cluster analysis were determined based on the maximum count value within the sedentary behavior category; MPA and VPA cut points were determined based on the minimum count values within these two PA intensity categories; LPA was further determined based on the sedentary behavior and MPA cut points.

### Statistical Analysis

The VM count for each epoch for each participant was categorized into a PA level based on each of the three sets of newly established wrist-worn accelerometer cut points and the hip reference, resulting in four separate PA level designations. Standard classification measures of sensitivity, specificity, false-negative rate, and false-positive rate were calculated to assess the discriminative ability of each method to correctly classify PA levels. Cohen kappa values were calculated to test the agreement between hip- and four wrist-derived measures, k-means, ROC, OLR, and Johansson’s cut points. Daily amount of time in each PA level was also calculated and compared against PA estimates from the hip-worn accelerometer. Univariate analysis of covariance was conducted to compare mean daily time in each PA intensity level for each of the wrist-worn ML-based cut points versus the hip reference and the Johansson’s cut points.

## Results

**Figure 3:**
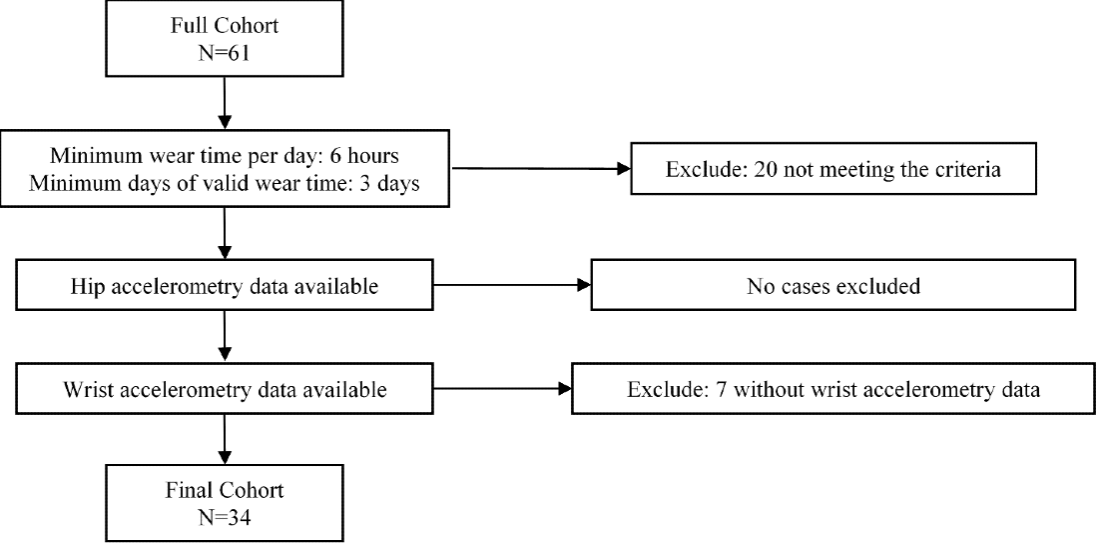
Data flow diagram.

All study participants were 3-5 years old, and more than 80% (29/34) of them were of Hispanic ethnicity (Table 1). Hip accelerometer identified 64.2% activity counts representing sedentary behavior and nearly 8.0% representing MVPA. Matched hip- and wrist-worn accelerometer data were collected and analyzed from 34 participants, yielding a total of 122,399 epochs (Figure 3).

**Table 1:** Descriptive characteristics of the study participants (N=34).

Variables		Total
Female, n (%)		20 (58.8)
Hispanic race, n (%)		29 (85.3)
Age (years), mean (SD)		3.97 (0.49)
Height (cm), mean (SD)		100.80 (4.70)
Weight (kg), mean (SD)		16.38 (2.157)
**Hip-based activity counts in each physical activity level, n** **(%)**		
	Sedentary behavior	78,538 (64.2)
	Light physical activity	34,243 (28.0)
	Moderate physical activity	6784 (5.5)
	Vigorous physical activity	2834 (2.3)
	Moderate-to-vigorous physical activity	9618 (7.8)

The three ML models grouped accelerometer counts differently and developed three sets of wrist-worn accelerometer VM cut points (lower and upper bounds; Table 2). For each ML model, mean count values increased as the PA intensity level increased, as expected.

**Table 2:** Wrist-worn accelerometer VM cut points established by each ML model and mean count value within each PA category.

Model		N		Cut points (cpm^a^)			Vector magnitude counts (cpm)	
					Lower Bound	Upper Bound		Mean (SD)
**Receiver operating characteristic** **analysis**								
	Sedentary behavior		70,848		0	3406		744.40 (1077.67)
	Light physical activity		20,194		3407	5690		4509.79 (652.33)
	Moderate physical activity		4009		5691	6219		5952.98 (150.75)
	Vigorous physical activity		27,348		6220	∞		10,054.39 (4267.24)
**Ordinal logistic regression model**								
	Sedentary behavior		92,143		0	5837		1629.59 (1900.25)
	Light physical activity		26,746		5838	14,020		8436.16 (2028.01)
	Moderate physical activity		1688		14,021	17,432		15,481.18 (968.54)
	Vigorous physical activity		1822		17,433	∞		22,350.31(4593.46)
**K-means analysis**								
	Sedentary behavior		62,815		0	2556		457.57 (759.38)
	Light physical activity		37,876		2557	7067		4655.99 (1270.28)
	Moderate physical activity		18,559		7068	14,535		9474.95 (1895.16)
	Vigorous physical activity		3149		14,536	∞		19,595.06 (4787.10)

^a^cpm: counts per minute.

### Agreement Between Each Wrist Method and the Hip Reference

Agreement between the wrist methods and the hip reference at epoch level is presented at Table 3. When grouping the MPA and VPA, the prediction accuracy of each wrist method was more comparable with the hip reference, based on a higher classification rate and kappa value.

**Table 3:** Agreement of each wrist cut point compared to the hip reference.

Activity intensity		Johansson Wrist	ROC^a^ analysis	OLR^b^ model	K-means analysis
**Sedentary behavior**					
	Sensitivity (%)	81.36	77.82	90.95	71.64
	Specificity (%)	72.77	77.82	52.78	85.07
	FPR (%)	27.23	22.18	47.22	14.93
	FNR (%)	18.64	22.18	9.05	28.36
	Kappa	0.53	0.54	0.47	0.53
**Light physical activity**					
	Sensitivity (%)	67.34	26.80	43.27	50.90
	Specificity (%)	75.69	87.50	86.47	76.81
	FPR (%)	24.31	12.50	56.73	23.19
	FNR (%)	32.66	73.20	13.53	49.10
	Kappa	0.39	0.16	0.32	0.27
**Moderate physical activity**					
	Sensitivity (%)	N/A^c^	4.47	8.83	48.42
	Specificity (%)	N/A	96.79	99.06	86.79
	FPR (%)	N/A	3.21	0.96	13.21
	FNR (%)	N/A	95.53	76.11	51.58
	Kappa	N/A	0.02	0.12	0.19
**Vigorous physical activity**					
	Sensitivity (%)	N/A	79.00	23.89	33.94
	Specificity (%)	N/A	79.00	99.04	98.17
	FPR (%)	N/A	21.00	0.96	1.83
	FNR (%)	N/A	21.00	76.11	66.06
	Kappa	N/A	0.11	0.28	0.31
**Moderate-to-vigorous physical activity**					
	Sensitivity (%)	16.02	78.89	24.27	68.37
	Specificity (%)	99.53	78.92	98.96	86.58
	FPR (%)	0.47	21.08	1.04	13.42
	FNR (%)	83.98	21.11	75.73	31.63
	Kappa	0.24	0.28	0.33	0.35
**Overall agreement**					
	Correct classification (%)	N/A	59.51	71.51	63.68
	Kappa	N/A	0.30	0.37	0.37
**Overall agreement when MPA and VPA were grouped together**					
	Correct classification (%)	72.30	63.63	72.37	65.58
	Kappa	0.45	0.35	0.39	0.40

^a^ROC: Receiver operating characteristic.

^b^OLR: Ordinal logistic regression.

^c^N/A: Not applicable.

According to the kappa values, all three ML models performed well on identifying sedentary behavior. When compared against the hip reference, ROC analysis and k-means cluster analysis derived cut points with acceptable sensitivity and specificity values (both >70%) and were comparable to the performance of Johansson et al [25] cut points.

In terms of classifying LPA, only the k-means LPA cut point resulted with sensitivity and specificity values greater than 50%. While the specificity value for k-means LPA cut point (76.81%) was similar to the Johansson cut point (75.69%), it had a much lower sensitivity value.

None of the three wrist methods were able to distinguish MPA as indicated by the low sensitivity and kappa values, although the k-means MPA cut point had the highest sensitivity (48.42%) and kappa value (0.19). OLR and k-means cut points also had low sensitivity values for identifying VPA (23.89% for OLR, 33.94 for k-means). Although the ROC cut point exhibited sensitivity and specificity values of 79%, the low kappa value (0.11) indicated that there was a low agreement between this method and the hip reference. When MPA and VPA were grouped together, the k-means cut points demonstrated higher sensitivity (68.37%), specificity (86.58%), and kappa values (0.35) than the other two wrist methods.

In general, k-means cut points resulted in sensitivity and specificity values above 50% for predicting sedentary behavior, LPA, and MVPA, with an acceptable kappa value for overall agreement (0.40).

### Physical Activity Estimates by Method

Table 4 presents daily amount of time and percent of time in each PA intensity level as assessed by the hip reference, Johansson et al [25] wrist VM cut points, and the three newly developed wrist methods. ROC and k-means sedentary behavior cut points were close to the hip reference on estimating sedentary behavior time. LPA estimates were similar among the Johansson et al [25] cut point, the hip reference, and the k-means wrist method. None of the three wrist methods were comparable to the hip reference on estimating MPA and MVPA. Univariate ANOVA showed a similar VPA estimates for the k-means wrist method and the hip reference.

**Table 4:** Daily time in each Physical Activity intensity level (%) by different sets of cut points.

Activity intensity	Hip reference		Johansson wrist		ROC^a^ analysis		OLR^b^ model		K-means analysis	
	Mean (SD)	%	Mean (SD)	%	Mean (SD)	%	Mean (SD)	%	Mean (SD)	%
Sedentary behavior	148.07 (32.15)	57.8^c^	194.81 (41.58)	63.9	157.95 (37.88)	58.5^c^	208.61 (33.65)	76.1	138.13 (37.63)	51.6^c^
Light physical activity	83.13 (25.71)	30.4^d^	100.84 (39.53)	34.6^d^	47.47 (12.97)	16.7	60.11 (26.45)	21.2	89.25 (23.68)	31.3^d^
Moderate physical activity	19.27 (10.95)	7.1	N/A	N/A	9.20 (3.44)	3.3	3.73 (2.18)	1.3	41.46 (19.87)	14.7
Vigorous physical activity	13.24 (20.74)	4.7^e^	N/A	N/A	60.84 (28.09)	21.5	3.82 (2.88)	1.4	6.74 (4.42)	2.4^e^
Moderate-to-vigorous physical activity	32.61 (30.78)	11.8	4.35 (3.16)	1.5	70.98 (61.63)	24.8	7.62 (4.77)	2.7	48.87 (23.47)	17.1

^a^ROC: Receiver operating characteristic.

^b^OLR: Ordinal logistic regression.

^c^ Indicates there is no statistical difference in PA estimate between the ML method and the hip-based reference for the Sedentary behavior intensity level. ^d^ Indicates there is no statistical difference in PA estimate between the ML method and the hip-based reference for the Sedentary behavior intensity level. ^e^ Indicates there is no statistical difference in PA estimate between the ML method and the hip-based reference for the Sedentary behavior intensity level.

## Discussion

This study applied wrist derived VM data to assess PA in preschoolers based on three ML models. In the supervised ML models, the ROC analysis and OLR model were able to distinguish VM counts into each PA intensity level by reading the intensity label of each epoch as assigned by the gold standard hip reference. As an unsupervised ML model, k-means cluster analysis successfully grouped accelerometer count values into four PA clusters. When examining the agreement and comparing PA estimates from the wrist data compared with the hip reference, the k-means cluster analysis had the best performance among three ML models tested. Additionally, the k-means method of assigning PA levels had better agreement with the hip reference than the previously published cut points developed by Johansson et al [25].

Our results showed that cut points derived from the k-means cluster analysis produced better agreement with the gold standard hip reference than the other two ML approaches across all sedentary behavior and PA intensity levels, as indicated by sensitivity, specificity, and kappa values. K-means sedentary behavior, LPA, and VPA cut points showed the highest similarity to the hip reference on estimating PA time. Additionally, the estimated percent time in sedentary behavior, LPA, and MVPA by the k-means wrist reference method was comparable to the findings by Jones et al [35], who adopted a hip-worn accelerometry protocol for assessing PA in preschoolers similar to the one used in this study.

One reason that the k-means method had superior performance could be that the use of unlabeled data for calibration may result in less biased cut points for classifying epochs into PA levels [36]. During the data training process for the supervised learning models, the hip reference-based PA levels were used as “labels” or “targets,” which the models are trying to use as a basis to classify each epoch into the “true PA state.” Since most of the epochs in our calibration data were observed in the sedentary state based on the hip reference, this could result in biasing the supervised model cut points toward lower activity levels. Thus, the lower activity levels might overly influence supervised learning models when developing cut points for wrist-worn accelerometers.

Similarly, the nature of the activities generating the calibration data may also influence the performance of the machine learning methods. For example, Butte et al [24] used 11 structured activities as the ground truth activity to calibrate the cut points for the hip reference [37]. However, others have found that using free-living activities for accelerometer calibration might generate higher counts per minute cut points than structured activities [38]. This might explain the higher MVPA cut point (≥16716 cpm) developed by Johansson et al [25], which incorporated free-play sessions during the calibration process, compared to our calibration data, which used only structured activities. Regardless, the k-means method showed superior performance in accurately assessing PA levels compared to the ROC, OLR and Johansson et al [25] methods. Thus, the k-means approach represents an improvement on existing methods for establishing wrist-based PA levels among pre-school aged children.

Our results showed that all wrist-based methods had difficulty in accurately assessing MPA. One possible explanation for this issue is that there is no consensus on what types of activities can represent MPA for this age group [25]. For example, the Johansson study [25] used a ball-toss activity, a 10-minute active video game, a 15-minute dancing activity, and an aerobic video activity to represent MPA, whereas Pate et al [39] defined the intensity between slow and brisk walking as the cut point for MPA and Sirard et al [40] used fast walking at 4.3 (SD 0.6) km/h as their criterion activity to represent MPA. Therefore, combined criterion-based and free-living activities for the generation of model training data may better reflect the full range of MPA, which could result in improved calibration processes for children in this age group. Future studies would also benefit from additional analysis of raw accelerometry data from wrist-worn devices with unsupervised learning methods.

### Limitations

This study has several limitations. First, participants in this study were from low-income minority families and, therefore, accelerometer counts collected from the study sample might not represent the activity patterns of the general population. Previous studies have shown that young children from low-income minority families had a significantly lower motor performance and are less active during a preschool day compared to children from higher income families [41,42]. However, previous research found no difference in cut points in older children and adults from different income and socioeconomic background [13]. Second, this study chose a 30 Hertz sampling rate for both hip and wrist accelerometer placement sites to collect raw acceleration data to make the results comparable to other studies, but this approach might lead to an underestimate of activity intensity [13]. For instance, Clevenger et al [43] found that greater sampling rate resulted in a higher estimation of high-intensity PA in preschoolers for both hip- and wrist-worn accelerometers, even though the difference was not significant. Thus, the study should be replicated using higher sampling rate magnitude. Another limitation is that this study collected data during school hours on 3 days, which may not reflect the general PA patterns of preschool-aged children. Hesketh et al [44] found that children were more active in daycare than at home and were more active during weekdays than weekends days. In this case, collecting accelerometry data from both weekdays and weekend days based on a 24-hour accelerometry protocol would reflect the PA pattern in this age group more accurately. Finally, the cut points from ML models were not validated against ground truth activities that can substantiate the accuracy of the wrist-derived data [16].

### Conclusion

This study demonstrated the potential of ML techniques to distinguish PA intensity levels, with the exception of MPA, in preschool-age children. Cut point established from k-means cluster analysis was comparable to the hip-reference criterion in predicting sedentary behavior. Although PA estimates from k-means cluster analysis of wrist-worn accelerometers were acceptable as compared to the hip reference, the finding needs to be replicated using ground truth activities in free-living setting.

## Abbreviations

LPA: light physical activity
ML: machine learning
MPA: moderate physical activity
MVPA: moderate-to-vigorous physical activity
OLR: ordinal logistic regression
PA: physical activity
ROC: receiver operating characteristic
VPA: vigorous physical activity
